# How mass sports events satisfy the “needs for a better life”: evidence from Chinese marathon runners’ psychological perceptions and support

**DOI:** 10.3389/fpsyg.2026.1765421

**Published:** 2026-03-26

**Authors:** Jiaxin Ma, Daoming Huang

**Affiliations:** 1 School of General Education, Deyang College of Urban Rail Transit, Deyang, China; 2 School of Physical Education, Southwestern University of Finance and Economics, Chengdu, China

**Keywords:** marathons, needs for a better life, participant perception, participant support, self-determination theory

## Abstract

**Introduction:**

Promoting well-being and quality of life through sports participation is a growing focus of research and policy worldwide. Mass sports events, such as marathons, serve as significant platforms for this purpose, yet the specific psychological mechanisms through which they foster participant support and well-being require deeper empirical exploration. Grounded in Self-Determination Theory (SDT), this research conceptualizes how marathon events satisfy participants’ psychological needs and examines the relationship between need satisfaction and supportive attitudes.

**Methods:**

A survey was conducted with 321 marathon participants in China. Exploratory factor analysis (using Principal Axis Factoring with Promax rotation) was first employed to validate the dimensional structure of need satisfaction perception. Subsequently, structural equation modeling, incorporating demographic variables as controls, was used to test the associations between the identified need dimensions and event support.

**Results:**

Exploratory factor analysis validated a three-dimensional structure of need satisfaction perception: (1) achievement-oriented, (2) city-support-oriented, and (3) social-bonding-oriented. Results from structural equation modeling indicated that all three dimensions were significantly and positively associated with event support, though with varying strengths. Achievement-oriented psychological needs perception showed the strongest association (*β* = 0.276, *p* < 0.001), followed by city-support-oriented (*β* = 0.194, *p* < 0.01) and social-bonding-oriented (*β* = 0.179, *p* < 0.01) perceptions. The model explained 21.6% of the variance in event support.

**Discussion:**

These findings provide a micro-psychological lens for understanding how sports events contribute to subjective well-being, offering empirical evidence that links the macro concept of “needs for a better life” with established psychological theory. The study offers practical insights for event organizers and city managers seeking to design experiences that address participants’ multi-faceted psychological needs, thereby enhancing engagement and fostering sustained public support.

## 1 Introduction

In contemporary society, there is a growing global emphasis on enhancing subjective well-being and quality of life beyond material prosperity. This shift underscores the importance of activities that fulfill higher-order psychological needs. In China, this societal aspiration is crystallized in the national discourse of meeting “the people’s growing needs for a better life,” which highlights comprehensive individual development and psychological fulfillment. Mass participation sports events have emerged as a significant cultural phenomenon in this context, serving not only as physical pursuits but also as potential platforms for experiencing achievement, social connection, and personal growth.

Marathon running exemplifies this trend, having witnessed explosive growth worldwide, particularly in China. According to the 2023 China Road Running Events Blue Book, 580 road running events were held nationwide, attracting 3.055 million participants, a figure that significantly surpasses pre-pandemic levels and underscores the event’ s massive appeal. Concurrently, supportive policies have been enacted to promote sports consumption and enrich the supply of such events, aligning with broader social goals. Despite their popularity and policy support, a critical question remains underexplored: How do marathon participants perceive the fulfillment of their fundamental psychological needs through the event experience, and how do these perceptions translate into their support for the event? Addressing this question is essential to move beyond descriptive accounts of motivation or external impacts, toward a mechanistic understanding of the participant–event relationship.

Extensive academic research has explored marathon participation, primarily focusing on participants’ motivations. Motivation plays a crucial role in sports, driving athletes’ behaviors and outcomes, encouraging them to set goals, and persisting in the face of challenges (Partyka and Waśkiewicz, 2024). Early studies emphasized physiological drivers like health improvement and weight management (Gerasimuk et al., 2021; Stempień et al., 2020), while subsequent work revealed the importance of psychological and social factors, such as personal achievement, self-esteem, and social identity (Waśkiewicz et al., 2018). Participation has also been linked to enhanced emotional well-being (Popov et al., 2019). Behind these motivations, Self-Determination Theory (SDT) points out that human behavior is driven by three basic psychological needs: autonomy, competence, and relatedness. The degree to which the environment supports these needs directly affects individuals’ motivation, sense of happiness, and sustained behavioral intentions (Ryan and Deci, 2000). However, most existing studies only describe the types of motivations, lacking the systematic integration of marathon participants’ multi-dimensional needs under the SDT framework, nor do they deeply examine the differential predictive effects of different need dimensions on their attitudes towards event support.

Another research direction focuses on the external impacts of marathon events, especially on the host cities. Studies have explored their benefits in enhancing the city image, promoting tourism consumption, and improving infrastructure (Gan et al., 2023; Huang et al., 2015). At the micro level, some studies have shown that events can enrich residents’ cultural lives and enhance their subjective sense of happiness (Huang et al., 2017; Zhang et al., 2020). However, such studies mainly adopt an external perspective, evaluating the impacts of events from the standpoint of the city or ordinary residents, while largely ignoring the internal perceptions of the participants themselves. There is less attention paid to the participants’ dual identities as “in-depth experiencers” and “urban citizens,” and how they internalize these external benefits and convert them into recognition and support for the events themselves. Although “needs for a better life” has been proposed as an important concept to measure people’s high-level needs, its operationalization and psychological mechanisms in the context of mass sports events remain to be explored.

Self-Determination Theory (SDT) posits that human behavior is driven by three basic psychological needs: autonomy, competence, and relatedness. This framework not only offers a mechanistic explanation for the diverse motivations of marathon participants but also elucidates how the external social environment shapes individual attitudes by fulfilling these needs. Thus, it helps bridge the gap between motivational micro-research and macro-level impact studies in the existing literature. In the context of marathon participation, SDT provides a coherent lens through which the macro social concept of “needs for a better life” can be reconceptualized. Specifically, participants’ complex experiences can be mapped onto the three basic needs: striving for a performance breakthrough aligns with competence; seeking belonging within running groups reflects relatedness; and perceiving that the urban environment supports one’s running hobby enhances autonomy. Accordingly, this study adopts marathon events as the research setting and employs SDT to operationalize participants’ “perception of needs for a better life” as a multi-dimensional construct consisting of achievement oriented, city support oriented, and social bonding oriented psychological needs. Through empirical investigation, we examine how each dimension influences participants’ support for the marathon event.

This study makes contributions in multiple aspects. At the theoretical level, it applies SDT to operationalize the micro-psychological theory of “a better life” within the marathon context, offering an integrated perspective that brings together insights from both motivation research and impact research in mass sports event studies. At the methodological level, it develops and provides initial validation for a multi-dimensional scale for measuring the perception of needs for a better life in this context, which may serve as a useful instrument for empirical testing in subsequent related studies. At the practical level, the research conclusions can provide evidence-based decision-making basis for event organizers and urban managers. By designing targeted events and urban environments that meet these basic psychological needs, they can effectively improve participants’ satisfaction and loyalty, and ultimately promote the healthy and sustainable development of mass sports events.

## 2 Theoretical framework and research hypotheses

### 2.1 Self-determination theory and the perception of needs for a better life

As a macro theory of human motivation and personality, Self-Determination Theory (SDT) provides a robust framework for understanding how individuals’ fundamental psychological needs are satisfied within specific social contexts (Ryan and Deci, 2000). The theory posits that humans are inherently driven by three basic and universal psychological needs: autonomy, competence, and relatedness. When these needs are supported by the environment and satisfied, individuals will experience a deeper sense of happiness, psychological growth, and behavioral persistence (Deci and Ryan, 2008). Marathon events, as large-scale, structured, and socially embedded activities, create a unique context that can profoundly impact participants’ psychological experiences. Grounded in SDT and aligning with the macro-social discourse on “a better life,” this study conceptualizes marathon participants’ perception of needs for a better life as the specific manifestation and satisfaction of these three basic psychological needs within the event context. The theoretical corresponding relationships between the three perception dimensions and the basic psychological needs of SDT are elaborated in detail below.

#### 2.1.1 Achievement-oriented psychological needs and competence

The achievement-oriented psychological needs perception refers to the positive experiences of accomplishment, mastery, and self-realization that participants derive from investing in and completing a marathon. This perception, stemming from the confirmation of personal capabilities and goal attainment, is a direct reflection of the SDT need for competence. Competence involves an individual’s inherent desire to feel effective and in control when interacting with their environment (Ryan and Deci, 2000). With its clear distance goals, strict cutoff times, and great physical and psychological challenges, marathon events naturally provide an ideal platform for participants to pursue and verify their competence (Masters and Ogles, 1995). From the accumulation of abilities in systematic pre-race training, to the real-time experience of effectiveness in overcoming the “wall” during the race, and finally to the sense of achievement and honor obtained by crossing the finish line, the entire participation process constitutes a complete cycle of competence need satisfaction (Reis et al., 2000). The resulting sense of pride and self-confidence has a strong intrinsic motivational effect on participants and is the core psychological driving force for runners to continue participating and investing deeply (Deci and Ryan, 2008). Therefore, this study conceptualizes the achievement-oriented psychological needs perception as the concentrated manifestation of participants’ need for competence within the marathon context.

#### 2.1.2 City-support-oriented psychological needs and autonomy

The city-support-oriented psychological needs perception captures the extent to which participants perceive that the host city’s development and transformations—triggered or accelerated by the marathon—create an environment that actively supports and validates their personal choice to engage in running. Theoretically, this dimension aligns with the SDT need for autonomy, defined as the desire to experience one’s behavior as self-endorsed, volitional, and congruent with personal values and interests (Ryan and Deci, 2000). In the context of a marathon, autonomy satisfaction extends beyond the initial decision to register. It is reinforced when participants observe tangible urban improvements, such as enhanced infrastructure, enriched public spaces, a more vibrant sports culture, and improved services, which signal the city’s commitment to fostering an active, healthy lifestyle. These changes collectively form a “need-supportive environment” that communicates to runners that their personal pursuit is recognized, facilitated, and valued by the city. When participants perceive that the city is not merely a passive backdrop but an active enabler of their running identity, they experience a deeper sense of volition and personal endorsement of their involvement. This environmental support transforms running from an individual hobby into a choice that is coherent with the evolving character of the city, thereby fulfilling the autonomy need in a socially embedded manner. Hence, this study conceptualizes city-support-oriented psychological needs perception as the satisfaction of autonomy through the perception of a supportive urban environment.

#### 2.1.3 Social-bonding-oriented psychological needs and relatedness

The social-bonding-oriented psychological needs perception encompasses participants’ positive evaluations of the social connections, community belonging, and broader social cohesion fostered by the marathon event. This dimension directly corresponds to the SDT need for relatedness. Relatedness is the innate desire to establish caring connections with others and to gain a sense of belonging (Baumeister and Leary, 1995). A marathon serves as a large-scale social platform that creates rich opportunities for such connection (Sheldon and Bettencourt, 2002). This is manifested at multiple levels: at the micro level, the camaraderie with running teammates and the mutual encouragement among runners on and off the track provide direct emotional support and a sense of belonging (Baumeister and Leary, 1995); at the macro level, as part of a grand urban event, participants will experience a strong sense of collective honor and group identity as social members. The perception that the event generates positive social benefits further satisfies participants’ need to be positively connected to a broader social group. Therefore, this study conceptualizes social-bonding-oriented psychological needs perception as the manifestation of relatedness need satisfaction within the specific social context of marathons.

### 2.2 Research hypotheses

This study aims to explore marathon participants’ perception of needs for a better life and its impact on event support. Based on Self-Determination Theory and combined with the specific context of marathon events, this study first puts forward hypotheses on the dimensional structure of the perception of needs for a better life, and then analyzes the relationships between each dimension and participants’ support.

Regarding the dimensional structure of the perception of needs for a better life. As a macro theory of human motivation, SDT posits that three basic psychological needs—autonomy, competence, and relatedness—are fundamental to psychological growth and well-being (Fonteyn et al., 2024). In the context of marathon events, these universal needs are externalized into specific perceptions: the experience of personal achievement, the perception of urban environmental support, and the satisfaction of social connections. Therefore, this study expects that participants’ perception of needs for a better life in marathons is a three-dimensional construct. Based on this, Hypothesis 1 is proposed:

Hypothesis 1: Marathon participants’ perception of needs for a better life comprises three distinct dimensions: achievement-oriented psychological needs perception, city-support-oriented psychological needs perception, and social-bonding-oriented psychological needs perception.

Regarding the impact of the achievement-oriented psychological needs perception on participants’ support. According to Goal Setting Theory, clear and challenging goals can stimulate individuals’ achievement motivation, and the achievement of goals will bring higher satisfaction (Sheldon and Bettencourt, 2002). The sense of achievement and effectiveness that marathon participants gain from completing extremely challenging events is their most direct and profound participation experience. This successful experience based on goal achievement will generate a strong intrinsic motivation, prompting individuals to have a high degree of recognition and loyalty to the events that can provide this successful experience. Therefore, this paper proposes Hypothesis 2:

Hypothesis 2: The achievement-oriented psychological needs perception has a significant positive impact on participants’ support for marathons.

Autonomy is satisfied when individuals perceive their actions to be volitional and supported by the environment (Ryan and Deci, 2000). When participants observe that hosting the marathon leads to tangible urban improvements, such as enhanced infrastructure, services, and sports culture, they perceive their personal choice to run as being validated and facilitated by the city. This positive perception of the host city will significantly affect their event satisfaction and support willingness (Koo et al., 2014). Therefore, this paper proposes Hypothesis 3:

Hypothesis 3: The city-support-oriented psychological needs perception has a significant positive impact on participants’ support for marathons.

Regarding the impact of the social-bonding-oriented psychological needs perception on participants’ support. Establishing and maintaining stable and positive interpersonal relationships is a basic human motivation (Baumeister and Leary, 1995). In marathon events, participants strengthen their social identity through interactions with runners, encouragement from the audience, and the experience of being members of the urban community. When individuals feel a strong sense of belonging and positive social ties within the event, they develop emotional attachment to it, which is a key driver of continued support and positive evaluation. Therefore, this paper proposes Hypothesis 4:

Hypothesis 4: The social-bonding-oriented psychological needs perception has a significant positive impact on participants’ support for marathons.

**Figure 1:**
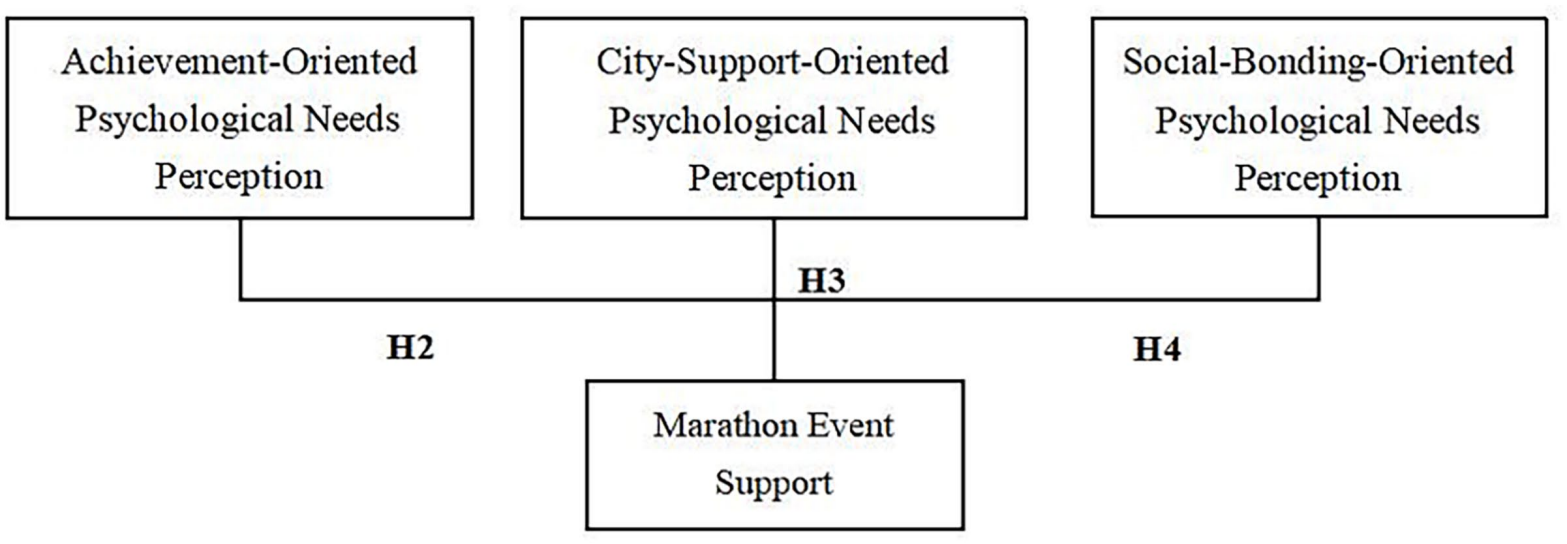
Participants’ perception of needs for a better life and event support in marathons.

The framework diagram of this paper is shown in Figure 1.

## 3 Methods

### 3.1 Measurements

This study employs a questionnaire survey method. The scale consists of two parts: the first part captures participants’ socio-demographic characteristics; the second part measures the core research variables, comprising four scales with a total of 21 items. These scales measure the three dimensions of perceived needs for a better life: achievement-oriented psychological needs perception, city-support-oriented psychological needs perception, and social-bonding-oriented psychological needs perception, along with the outcome variable of event support.

#### 3.1.1 Participants’ perception of marathon event participation

For the measurement of participants’ perception of marathon event participation, referring to the mature scales of PNSE (Psychological Need Satisfaction in Exercise) and SSRS (Social Support Rating Scale), relevant literature on the impact of sports events on host cities, and combining the connotation of needs for a better life in the new era, questionnaire items are designed. The questionnaire is structured into three dimensions for perceived needs (17 items): achievement-oriented, city-support-oriented, and social-bonding-oriented. All items for these dimensions are measured on a 5-point Likert scale (1 = “Strongly Disagree,” 5 = “Strongly Agree”). Specifically, for achievement-oriented psychological needs perception, it mainly refers to the Psychological Need Satisfaction in Exercise Scale (PNSE), which measures the satisfaction of psychological needs in sports based on Self-Determination Theory (Wilson et al., 2006); for social-bonding-oriented psychological needs perception, the measurement items mainly refer to the Social Support Rating Scale (SSRS) (Shuiyuan, 1994); for city-support-oriented psychological needs perception, the measurement items are mainly drawn from relevant studies on the impact of sports events on host cities (Chen, 2011; Wang et al., 2020).

#### 3.1.2 Participants’ event support

For the measurement of event support, referring to the literature such as Prayag et al. (2013) and Lian and Huang (2017), 4 items are set from two dimensions: emotional and behavioral tendency components, namely “I hope marathon events will continue to be held in the future,” “I hope to have more opportunities to participate in marathon events,” “I will recommend marathon events to my family or friends around me,” and “I am willing to pay a reasonable fee to participate in marathon events.” The above questions are all measured using a 5-point Likert scale. Table 1 shows the structure of the questionnaire.

**Table 1:** Information of measurement scales for research variables.

Variable name	Items	Scale source
Achievement-oriented psychological needs perception	I can decide the pace during the race independently	PNSE scale (Wilson et al., 2006)
	I can decide whether to warm up before the race independently	
	I believe I can complete the entire distance of the marathon	
	I am satisfied with my physical fitness level	
	Even if I encounter difficulties during the race, I can persist in finishing the entire course	
City-support-oriented psychological needs perception	Through the marathon event, I feel that the city has gained considerable economic benefits (such as tourism, consumption, employment opportunities)	Wang et al. (2020) and Chen (2011)
	Through the marathon event, I feel that the city’s international popularity and image have been improved	
	Through the marathon event, I have felt the unique cultural and historical characteristics of the city	
	Through the marathon event, I have felt the planning and transformation of the city in terms of roads, public transportation, leisure facilities, etc.	
	Through the marathon event, I feel that the government has performed transparently and credibly in event organization, information release, and logistics support	
	Through the marathon event, I feel that local residents’ awareness of exercise, health concepts, and willingness to participate in sports activities have been improved	
Social-bonding-oriented psychological needs perception	Before and after the race, I received practical help and emotional encouragement from my family or friends	SSRS scale (Shuiyuan, 1994)
	On the track, I received practical help such as directions, supplies, or first aid from volunteers	
	On the track, I felt encouragement and help from other runners	
	After the race, I can obtain continuous support from organizations such as communities or running groups (such as recovery training resources)	
	I often participate in running club or community activities to obtain emotional and information support	
	Participating in the event makes me feel that I am a member of the general runner community	
Event support	I hope marathon events will continue to be held in the future	Prayag et al. (2013) and Lian and Huang (2017)
	I hope to have more opportunities to participate in marathon events	
	I will recommend marathon events to my family or friends around me	
	I am willing to pay a reasonable fee to participate in marathon events	

### 3.2 Data collection

Data were collected through a combination of online and offline channels to access a broad spectrum of marathon participants. The online survey was distributed via hyperlinks posted in major Chinese running communities and social media groups (WeChat running groups, and forums on running apps Keep). Offline, paper questionnaires were administered at the finish-line areas of two large-scale marathon events in China (e.g., Chengdu Marathon and Beijing Marathon) in the same year, where researchers invited participants to complete the survey immediately after finishing the race.

To ensure data quality, several screening rules were applied before analysis. Questionnaires were deemed invalid and excluded if: (1) the completion time was less than 120 s (based on pilot testing); (2) responses showed obvious straight-lining (identical answers across all items); or (3) answers to reverse-coded attention-check items (if included) were inconsistent.

A total of 360 questionnaires were collected. After applying the above exclusion criteria, 321 valid responses were retained, yielding an effective response rate of 89.2%. All participants were informed of the academic purpose of the study, assured of anonymity and confidentiality, and provided informed consent before proceeding. Data were analyzed using SPSS 26.0 and AMOS for subsequent statistical testing.

## 4 Results

### 4.1 Descriptive statistics

Table 2 shows the demographic characteristics of the valid samples. The sample of this study (*N* = 321) is evenly distributed in terms of gender. The age structure is centered on young and middle-aged runners aged 26–40 (accounting for 60.75% in total), which is consistent with the characteristics of the main group of marathon runners. The educational level of the sample is mainly secondary education, and the occupations are mostly freelancers and students. The vast majority of respondents have multiple marathon participation experiences (62.31% have participated 4 times or more), ensuring that they have a relatively mature cognition and perception of the event.

**Table 2:** Demographic statistics results.

Name	Options	Frequency	Percentage %
Gender	Male	167	52.025
	Female	154	47.975
Age	Under 18	4	1.246
	18~25	29	9.034
	26–30	84	26.168
	31~40	111	34.579
	41~50	59	18.380
	51~60	27	8.411
	Over 60	7	2.181
Educational level	Junior high school or below	68	21.184
	Senior high school/technical secondary school/technical school	253	78.816
	College diploma	135	42.056
	Bachelor’s degree	102	31.776
	Master’s degree or above	48	14.953
Occupation	Employed (enterprise)	18	5.607
	Employed (institution/government)	18	5.607
	Freelancer	155	48.287
	Student	110	34.268
	Others	56	17.445
Marathon experience	1–3 times	98	30.530
	4–5 times	139	43.302
	More than 5 times	61	19.003

### 4.2 Reliability test

Cronbach’s alpha coefficient is an indicator for testing questionnaire reliability, which is widely used in the analysis of empirical data. This study also uses corrected item-total correlation (CITC) to measure the reliability of individual question items. Table 3 shows the results of the reliability analysis. The Cronbach’s alpha coefficients of the four dimensions of achievement-oriented psychological needs perception, city-support-oriented psychological needs perception, social-bonding-oriented psychological needs perception, and event support are 0.875, 0.901, 0.900, and 0.839, respectively. The corrected item-total correlation of all items is greater than 0.4, and deleting any item will not lead to an increase in the alpha coefficient. In summary, the measurement tool of this study has good reliability, and the data is stable and reliable.

**Table 3:** Reliability test.

Construct	Items	Corrected item-total correlation	Cronbach’s alpha if deleted	Cronbach’s alpha
Achievement-oriented psychological needs perception	Q1	0.721	0.845	0.875
	Q2	0.690	0.853	
	Q3	0.722	0.845	
	Q4	0.709	0.848	
	Q5	0.682	0.854	
City-support-oriented psychological needs perception	Q6	0.749	0.880	0.901
	Q7	0.721	0.884	
	Q8	0.753	0.880	
	Q9	0.703	0.887	
	Q10	0.718	0.885	
	Q11	0.735	0.882	
Social-bonding-oriented psychological needs perception	Q12	0.713	0.885	0.900
	Q13	0.728	0.882	
	Q14	0.745	0.880	
	Q15	0.757	0.878	
	Q16	0.719	0.884	
	Q17	0.706	0.886	
Event support	Q18	0.678	0.795	0.839
	Q19	0.648	0.807	
	Q20	0.681	0.793	
	Q21	0.682	0.792	

### 4.3 Validity test and exploratory factor analysis

First, analyze whether the research data is suitable for factor analysis. Table 4 shows the results of the KMO and Bartlett’s test. It can be seen from the table that the KMO value is 0.916, which is greater than 0.6, meeting the prerequisite for factor analysis, indicating that the data can be used for factor analysis research. In addition, the data passes the Bartlett’s test of sphericity (*p* < 0.05), indicating that the research data is suitable for factor analysis.

**Table 4:** KMO and Bartlett’s test.

Measure		Value
KMO value		0.916
Bartlett’s test of sphericity	Approx. chi-square	3641.409
	df	210.000
	*p*-value	0.000

Exploratory factor analysis was conducted using Principal Axis Factoring with Promax oblique rotation, which allows for correlations among factors. As presented in Table 5, four factors with eigenvalues greater than 1 were extracted, with initial eigenvalues of 7.672, 2.523, 2.101, and 1.816, respectively. Following extraction, these four factors jointly accounted for 59.47% of the total variance. Due to the oblique rotation method employed, the variance explained by each rotated factor was redistributed to 34.62, 10.13, 8.07, and 6.66%, respectively. It should be noted that under oblique rotation, the variance percentages of individual factors are not additive, and the total cumulative variance remains 59.47%. These results provide initial support for Hypothesis 1, indicating that marathon participants’ perception of needs for a better life, together with their event support, exhibits a clear four-factor structure consistent with the theoretically proposed dimensions of achievement-oriented, city-support-oriented, social-bonding-oriented psychological needs perceptions, and event support.

**Table 5:** Total variance explained.

Factor	Initial Eigenvalues			Extraction sums of squared loadings			Rotation sums of squared loadingsa
	Total	% of variance	Cumulative %	Total	% of variance	Cumulative %	Total
1	7.672	36.535	36.535	7.27	34.62	34.62	5.321
2	2.523	12.016	48.551	2.127	10.129	44.749	5.096
3	2.101	10.005	58.556	1.694	8.065	52.814	4.942
4	1.816	8.648	67.204	1.398	6.656	59.47	3.929
5	0.613	2.921	70.124				
6	0.588	2.799	72.923				
7	0.533	2.536	75.46				
8	0.519	2.473	77.933				
9	0.47	2.239	80.172				
10	0.452	2.153	82.325				
11	0.424	2.02	84.345				
12	0.411	1.955	86.301				
13	0.391	1.86	88.16				
14	0.366	1.742	89.902				
15	0.347	1.651	91.553				
16	0.343	1.632	93.185				
17	0.328	1.56	94.745				
18	0.313	1.492	96.237				
19	0.285	1.357	97.595				
20	0.272	1.298	98.892				
21	0.233	1.108	100				

aMeans that when factors are correlated (oblique rotation), sums of squared loadings cannot be added to obtain a total variance.

Table 6 presents the factor loadings matrix resulting from the Promax oblique rotation. All items loaded highly on their theoretically intended factors, with factor loadings exceeding 0.70, while cross-loadings on other factors were generally below 0.40. Specifically, Factor 1 showed high loadings on items Q6 to Q11 (0.746–0.806), corresponding to city-support-oriented psychological needs perception; Factor 2 showed high loadings on items Q12 to Q17 (0.750–0.809), corresponding to social-bonding-oriented psychological needs perception; Factor 3 showed high loadings on items Q1 to Q5 (0.738–0.784), corresponding to achievement-oriented psychological needs perception; and Factor 4 showed high loadings on items Q18 to Q21 (0.723–0.769), corresponding to event support. These results demonstrate satisfactory convergent and discriminant validity of the measurement scale, confirming that the four-factor structure aligns well with the theoretical framework.

**Table 6:** Factor loadings matrix.

Items	Factor			
	1	2	3	4
Q1	0.409	0.327	0.783	0.316
Q2	0.335	0.329	0.748	0.343
Q3	0.358	0.355	0.784	0.314
Q4	0.387	0.354	0.772	0.362
Q5	0.389	0.271	0.738	0.35
Q6	0.798	0.385	0.413	0.333
Q7	0.764	0.31	0.393	0.308
Q8	0.806	0.305	0.374	0.25
Q9	0.746	0.295	0.382	0.308
Q10	0.765	0.279	0.359	0.311
Q11	0.78	0.362	0.375	0.351
Q12	0.322	0.755	0.325	0.267
Q13	0.314	0.773	0.332	0.285
Q14	0.278	0.799	0.294	0.25
Q15	0.365	0.809	0.383	0.341
Q16	0.309	0.763	0.324	0.321
Q17	0.339	0.75	0.354	0.26
Q18	0.339	0.239	0.327	0.758
Q19	0.31	0.265	0.338	0.723
Q20	0.264	0.311	0.311	0.765
Q21	0.299	0.304	0.371	0.769

### 4.4 Confirmatory factor analysis

To further examine the structural validity of the measurement model, a confirmatory factor analysis was conducted using AMOS 26.0 on the four-factor model comprising achievement-oriented, city-support-oriented, social-bonding-oriented psychological needs perceptions, and event support. Model parameters were estimated using the maximum likelihood method. As shown in Table 7, all model fit indices met or exceeded commonly accepted thresholds: the chi-square to degrees of freedom ratio (CMIN/DF) was 1.188, below the stringent criterion of 3; the normed fit index (NFI = 0.942), relative fit index (RFI = 0.933), incremental fit index (IFI = 0.990), Tucker–Lewis index (TLI = 0.989), comparative fit index (CFI = 0.990), and goodness-of-fit index (GFI = 0.940) all exceeded the recommended value of 0.90; and the root mean square error of approximation (RMSEA) was 0.024, well below the cutoff of 0.08. These results indicate that the hypothesized four-factor measurement model fits the data well, demonstrating satisfactory construct validity and warranting further structural equation modeling analysis. Convergent validity was evaluated by examining factor loadings, composite reliability (CR), and average variance extracted (AVE) for each construct.

**Table 7:** CFA.

Fit index	CMIN	DF	CMIN/DF	NFI	RFI	IFI	TLI	CFI	GFI	RMSEA
Result	217.454	183.000	1.188	0.942	0.933	0.990	0.989	0.990	0.940	0.024
Criterion			<3	>0.9	>0.9	>0.9	>0.9	>0.9	>0.9	<0.08

As shown in Table 8 (Convergent Validity Results), all standardized factor loadings for the items were statistically significant (*p* < 0.001) and exceeded the recommended threshold of 0.70, indicating that each item adequately reflected its corresponding latent construct. The composite reliability (CR) values for achievement-oriented (0.876), city-support-oriented (0.901), social-bonding-oriented (0.900), and event support (0.840) all exceeded the recommended criterion of 0.70, demonstrating good internal consistency. Furthermore, the average variance extracted (AVE) for each construct ranged from 0.567 to 0.603, meeting or approaching the recommended threshold of 0.50, which suggests satisfactory convergent validity.

**Table 8:** Convergent validity results.

Construct	Item	Unstandardized factor loading	S.E.	C.R.	Standardized factor loading	AVE	CR
Achievement-oriented	Q1	1.000			0.785	0.585	0.876
	Q2	0.878	0.064	13.705	0.747		
	Q3	1.006	0.070	14.369	0.779		
	Q4	0.996	0.070	14.227	0.772		
	Q5	0.947	0.070	13.528	0.739		
City-support-oriented	Q6	1.000			0.802	0.603	0.901
	Q7	0.907	0.061	14.871	0.766		
	Q8	0.962	0.062	15.586	0.795		
	Q9	0.802	0.055	14.455	0.749		
	Q10	0.894	0.061	14.710	0.760		
	Q11	0.927	0.060	15.326	0.785		
Social-bonding-oriented	Q12	1.000			0.756	0.601	0.900
	Q13	0.994	0.071	13.975	0.772		
	Q14	1.036	0.072	14.314	0.789		
	Q15	1.109	0.075	14.806	0.813		
	Q16	1.019	0.073	13.868	0.767		
	Q17	0.962	0.071	13.572	0.752		
Event support	Q18	1.000			0.758	0.567	0.840
	Q19	0.998	0.082	12.184	0.725		
	Q20	1.084	0.085	12.771	0.763		
	Q21	1.071	0.083	12.842	0.767		

Discriminant validity was assessed by comparing the square root of the AVE for each construct with the correlations between constructs. As presented in Table 9 (Pearson Correlations and Square Roots of AVE), the square root of the AVE for each construct (shown in bold on the diagonal) was greater than its highest correlation with any other construct. Specifically, the square roots of AVE for achievement-oriented (0.765), city-support-oriented (0.777), social-bonding-oriented (0.775), and event support (0.753) all exceeded their respective inter-construct correlations, which ranged from 0.385 to 0.500. These results provide strong evidence of discriminant validity, indicating that the four constructs are empirically distinct from one another. Additionally, all constructs were positively and significantly correlated (*p* < 0.001), which aligns with the theoretical expectation that the three psychological needs are interrelated while remaining conceptually separate.

**Table 9:** Pearson correlations and square roots of AVE.

Construct	Achievement-oriented	City-support-oriented	Social-bonding-oriented	Event support
Achievement-oriented	0.765			
City-support-oriented	0.500***	0.777		
Social-bonding-oriented	0.441***	0.431***	0.775	
Event support	0.452***	0.409***	0.385***	0.753

***p < 0.001.

### 4.5 Correlation analysis

To explore the impact of each factor of the needs for a better life scale on the participants’ support factor, this paper uses correlation analysis and regression analysis to explore the relationships between the three major factors of participants’ perception of needs for a better life and the participants’ support factor. Table 10 shows the results of the Pearson correlation coefficient analysis. The results show that the three independent variables (achievement-oriented, city-support-oriented and social-bonding-oriented psychological needs perception) are all significantly positively correlated with the dependent variable (event support). In addition, the three independent variables are also significantly positively correlated with each other. This result provides preliminary evidence for examining the predictive effect of needs perception on event support in the subsequent regression analysis.

**Table 10:** Pearson correlation matrix.

Construct	Mean	SD	Achievement-oriented psychological needs perception	City-support-oriented psychological needs perception	Social-bonding-oriented psychological needs perception	Event support
Achievement-oriented psychological needs perception	3.579	0.991	1.000			
City-support-oriented psychological needs perception	3.550	1.018	0.444**	1.000		
Social-bonding-oriented psychological needs perception	3.549	0.995	0.390**	0.387**	1.000	
Event support	3.625	0.939	0.389**	0.357**	0.334**	1.000

All correlation coefficients are significant at **p < 0.001.

### 4.6 Structural equation modeling analysis

Prior to examining the path coefficients, the overall fit of the structural model was assessed. As shown in Table 11 (Confirmatory Factor Analysis Model Fit), the model fit indices indicated a good fit between the hypothesized model and the empirical data: the chi-square to degrees of freedom ratio (CMIN/DF) was 1.269, below the recommended threshold of 3; the normed fit index (NFI = 0.931), relative fit index (RFI = 0.915), incremental fit index (IFI = 0.978), Tucker–Lewis index (TLI = 0.971), comparative fit index (CFI = 0.975), and goodness-of-fit index (GFI = 0.931) all exceeded the recommended value of 0.90; and the root mean square error of approximation (RMSEA) was 0.035, well below the cutoff of 0.08. These results demonstrate that the structural model exhibits satisfactory fit, warranting further interpretation of the path coefficients.

**Table 11:** Model fit indices for confirmatory factor analysis.

Fit index	CMIN	DF	CMIN/DF	NFI	RFI	IFI	TLI	CFI	GFI	RMSEA
Result	232.154	183.000	1.269	0.931	0.915	0.978	0.971	0.975	0.931	0.035
Criterion			<3	>0.9	>0.9	>0.9	>0.9	>0.9	>0.9	<0.08

Table 12 presents the path coefficient estimates of the structural equation model. After controlling for demographic variables including gender, age, education level, occupation, marathon experience, and monthly income, the model tested the effects of the three core independent variables on event support. Achievement-oriented psychological needs perception had a significant positive effect on event support (*β* = 0.276, *p* < 0.001), supporting H2. City-support-oriented psychological needs perception had a significant positive effect on event support (*β* = 0.194, *p* < 0.01), supporting H3. Social-bonding-oriented psychological needs perception had a significant positive effect on event support (*β* = 0.179, *p* < 0.01), supporting H4. All three standardized path coefficients were positive and statistically significant, with achievement-oriented need perception exhibiting the strongest predictive power (*β* = 0.276), followed by city-support-oriented (*β* = 0.194) and social-bonding-oriented (*β* = 0.179). These findings are consistent with the regression results, further confirming the robustness of the research hypotheses.

**Table 12:** Path coefficient test results of the structural equation model.

Path			Unstandardized path coefficient	S.E.	C.R.	*p*	Standardized path coefficient
Event support	←	Achievement-oriented	0.238	0.064	3.69	***	0.276
Event support	←	City-support-oriented	0.153	0.056	2.705	**	0.194
Event support	←	Social-bonding-oriented	0.161	0.062	2.605	**	0.179
Event support	←	Gender	0.021	0.035	0.854	0.245	0.02
Event support	←	Age	0.052	0.037	1.211	0.175	0.051
Event support	←	Education level	0.018	0.036	0.758	0.387	0.016
Event support	←	Occupation	0.087	0.034	1.574	0.088	0.085
Event support	←	Marathon experience	0.028	0.031	0.957	0.211	0.027
Event support	←	Monthly income	0.011	0.032	0.711	0.574	0.010

Significance levels are indicated as follows: **p < 0.01, **p < 0.001.

None of the control variables (gender, age, education, occupation, marathon experience, or income) had a statistically significant effect on event support (all *p* > 0.05). This indicates that the predictive effects of the core variables are robust and not confounded by these demographic characteristics. In summary, even after accounting for potential confounding factors, the three dimensions of marathon participants’ “needs for a better life” perception remain independent and significant positive predictors of their event support intention.

### 4.7 Common method bias test

Given that all data were collected through self-report questionnaires at a single time point, there was a potential risk of common method bias. To assess this concern, Harman’s single-factor test was conducted by loading all measurement items into an unrotated exploratory factor analysis. The results showed that the first factor accounted for 36.535% of the total variance, which is below the commonly recommended threshold of 40% (or 50%). This indicates that no single factor explained the majority of the variance, suggesting that common method bias is unlikely to be a serious concern in this study.

## 5 Discussion

Based on Self-Determination Theory, this study explores the impact of marathon participants’ perception of needs for a better life on their event support. Through the data analysis of 321 valid questionnaires, the research model is well supported. The research results reveal the internal psychological mechanism through which mass sports events meet people’s yearning for a better life.

### 5.1 Theoretical interpretation of research findings

Grounded in Self-Determination Theory, this study examined how marathon participants’ perception of needs for a better life relates to their supportive attitudes toward the event. The findings offer several theoretical insights.

First, the results provide empirical support for operationalizing the macro-social concept of “needs for a better life” within the specific domain of marathon participation as a three-dimensional structure derived from SDT. The clear factor structure identified through exploratory and confirmatory factor analyses confirms that participants’ diverse experiences can be meaningfully mapped onto achievement-oriented (competence), city-support-oriented (autonomy), and social-bonding-oriented (relatedness) need perceptions. This operationalization offers a conceptual bridge between a high-level social discourse and an established micro-theory of motivation, addressing a gap in the literature where macro-impact analyses and micro-motivational studies have often proceeded separately.

Second, the structural equation model results, obtained after controlling for demographic variables, indicate that all three need-perception dimensions are significantly and positively associated with event support. The model explains 21.6% of the variance in participants’ supportive attitudes, suggesting that while psychological need satisfaction is a meaningful contributor, other factors not captured in this study—such as event service quality, perceived value, or prior event experiences—may also play important roles. Nevertheless, the significant associations observed align with SDT’s core proposition: social contexts that support individuals’ basic psychological needs tend to foster positive attitudes and behavioral intentions. Marathon events, as a complex and socially embedded context, appear to cultivate participant support in part by addressing these multi-level psychological needs.

Third, the differential strengths of the three pathways offer a more nuanced understanding of how need satisfaction operates in this setting. The finding that achievement-oriented need perception exhibits the strongest association with event support underscores the centrality of competence satisfaction in goal-directed athletic pursuits. The significant roles of city-support-oriented and social-bonding-oriented need perceptions further suggest that autonomy satisfaction can be enhanced by perceived environmental support, and that relatedness satisfaction contributes through the sense of community fostered by the event. Together, these findings extend the application of SDT to the context of mass-participation sports events, illustrating how a well-designed event can serve as a need-supportive environment that cultivates participant engagement and support.

### 5.2 Differential analysis of the impacts of each dimension and its significance

The core finding of this study is that the three need perception dimensions have differential impact paths on event support.

#### 5.2.1 The core driving role of achievement-oriented psychological needs perception

This dimension shows the strongest predictive power. The challenging nature of marathons makes them an excellent stage to verify and enhance personal competence. From arduous training to completing the race, the sense of effectiveness, control, and the ultimate great sense of achievement accumulated throughout the process constitute the participants’ most direct and profound core experience. This intrinsic passion and satisfaction are the most powerful intrinsic motivations driving their supportive behaviors. This indicates that alongside peripheral services, the event’s core ability to provide participants with an experience of challenging themselves, proving their abilities, and gaining a sense of achievement is a primary driver for sustaining their support.

#### 5.2.2 The synergistic supporting role of city-support-oriented psychological needs perception and social-bonding-oriented psychological needs perception

Both city-support-oriented and social-bonding-oriented need perceptions also had significant positive associations with event support, although the effects are slightly weaker. This indicates that the satisfaction of participants’ autonomy needs does not only come from the freedom to choose to participate in the event personally but also from their perception that the urban environment where they live supports and empowers their hobbies. When participants see that the city has become better and more dynamic due to the event, they will feel that their personal values are consistent with the city’s development direction. This “perception of environmental support” greatly strengthens their sense of autonomy and transforms into dual recognition and pride for the event and the city. Similarly, the significance of social-bonding-oriented psychological needs perception highlights the social attribute of marathons. The sense of community belonging created by the event, the camaraderie among runners, and the collective honor of being a member of the social community effectively meet the participants’ need for relatedness.

### 5.3 Research implications

These findings offer actionable insights for event organizers and city managers seeking to enhance participant support through need-supportive event design.

First, given that achievement-oriented need perception emerged as the strongest predictor, organizers should prioritize optimizing participants’ sense of personal accomplishment. This can be achieved by providing detailed performance feedback, creating memorable finish-line rituals, and designing the event to highlight the progressive mastery of the marathon challenge.

Second, the significance of city-support-oriented need perception suggests that events should be designed to make urban support tangible. Integrating the city’s landmarks and culture into the event narrative, and visibly communicating how the marathon contributes to urban improvements, can help participants perceive their running hobby as being valued and facilitated by the city, thereby strengthening their sense of autonomy.

Third, the positive role of social-bonding-oriented need perception highlights the importance of fostering community. Creating platforms for ongoing interaction among participants—such as official running clubs or post-event social gatherings—can transform ephemeral race-day camaraderie into lasting social ties, deepening emotional attachment to the event.

## Data Availability

The original contributions presented in the study are included in the article/Supplementary material, further inquiries can be directed to the corresponding author.
